# Mediterranean spotted fever: clinical and laboratory characteristics of 415 Sicilian children

**DOI:** 10.1186/1471-2334-6-60

**Published:** 2006-03-22

**Authors:** Claudia Colomba, Laura Saporito, Valentina Frasca Polara, Raffaella Rubino, Lucina Titone

**Affiliations:** 1 Istituto di Patologia Infettiva e Virologia, Università di Palermo, Piazza Montalto 8, 90134 Palermo, Italy

## Abstract

**Background:**

Mediterranean spotted fever (MSF) is an acute febrile, zoonotic disease caused by *Rickettsia conorii *and transmitted to humans by the brown dogtick *Rhipicephalus sanguineus*. Nearly four hundred cases are reported every year (mainly from June to September) on the Italian island of Sicily. The aim of the study was to analyze the clinical and laboratory characteristics of patients with MSF and the efficacy of the drugs administered.

**Methods:**

Our study was carried out on 415 children with MSF, during the period January 1997 – December 2004, at the "G. Di Cristina" Children's hospital in Palermo, Sicily, Italy. On admission patients' clinical history, physical and laboratory examination and indirect immunofluorescence antibody test (IFAT) for *Rickettsia conorii *were performed. Diagnosis was considered confirmed if the patients had an MSF diagnostic score greater than or equal to 25 according to the Raoult's scoring system. All patients were treated with chloramphenicol or with macrolides (clarithromycin or azithromycin).

**Results:**

Fever, rash and tache noire were present in 386 (93%), 392 (94.5%) and 263 (63.4%) cases respectively. Eighteen (4.6%) children showed atypical exanthema. Chloramphenicol and newer macrolides all appeared to be effective and safe therapies.

**Conclusion:**

Clinical features of 415 children with MSF were similar to those reported by other authors except for a lower incidence of headache, arthralgia and myalgia and a higher frequency of epato-splenomegaly. Concerning therapy, clarithromycin can be considered a valid alternative therapy to tetracyclines or chloramphenicol especially for children aged < eight years.

## Background

Mediterranean spotted fever (MSF) is an acute febrile, zoonotic disease caused by *Rickettsia conorii *and transmitted to humans by the brown dog tick *Rhipicephalus sanguineus*, which is generally considered to be the most common tick in the Mediterranean area. Nearly four hundred cases are reported every year (mainly from June to September) on the Italian island of Sicily [1]. Interestingly, the number of cases in Italy and elsewhere appears to have increased over the past 20 years. Sporadic cases have also been diagnosed in travelers to other countries, and, in North America, MSF is one of the most frequently imported types of rickettsioses [2]. Until recently *Rickettsia conorii *was thought to be the main species in Europe. However, in the last decade newly recognized tick-borne rickettsioses have been shown to be prevalent in Europe; in 1996 and 1997, the first documented cases of infection due to *Rickettsia mongolotimonae *and *Rickettsia slovaca *respectively, were reported in France [3,4]. More recently *Rickettsia helvetica *has been identified in Sweden and France [5,6] and *Rickettsia Israeli *in Sicily and Portugal [7,8]. In Sicily two subspecies of Rickettsia conorii has been found: *Rickettsia conorii conorii *and *Rickettsia Israeli *[9]. MSF is typically characterized by fever, skin rash and a black eschar at the site of the tick bite ("tache noire") [10]. Actually more and more frequently severe forms are described and the current fatality rate of MSF is increasing [11]. Diagnosis is based on epidemiological, clinical and laboratory criteria [12]. Serological confirmation of infection is obtained using indirect immunofluorescence against *Rickettsia conorii*. Promptly administered antibiotic treatment shortens the symptomatic period of MSF infection and prevents the appearance of severe complications. Over the last few years the new macrolides have been widely employed and have became the antibiotic of choice for treating pediatric MSF [13,14].

The objective of this study was to analyze the clinical and laboratory characteristics of patients diagnosed with MSF at our hospital over the last eight years and to analyze the efficacy of the drugs administered.

## Methods

All patients diagnosed with MSF at the "G. Di Cristina" Children's hospital in Palermo, Italy, during the period January 1997 – December 2004, were included in this case series. Clinical suspicion of MSF was based on a history of a tick bite or direct contact with dogs, and on the presence of fever, non confluent maculopapular rash, tache noire, lymphadenopathy. On admission, a complete physical and laboratory examination (complete blood cell count, kidney and liver function tests, determination of the erythrocyte sedimentation rate, urine analysis) was carried out on most children. An indirect immunofluorescence assay for *Rickettsia conorii *was performed using a commercial kit (*Rickettsia conorii*-Spot; bio-Merieux, Marcy l'Etoile, France) when the first blood sample was taken and then, again, two weeks later (acute and convalescent-phase serum samples). Diagnosis was considered confirmed if the patients had an MSF diagnostic score of ≥ 25 according to the scoring system described in Raoult et al. [12].

All patients were treated with chloramphenicol or with macrolides. Chloramphenicol, administered orally or intravenously at a dosage of 50 mg/Kg/day in four divided doses for 7 days, was the first choice therapy until 1999. Since 2000, clarithromycin or azithromycin have been administered orally at a dosage of 15 mg/Kg/day in two divided doses for 7 days and 10 mg/Kg once daily for 3 days, respectively. Intravenously administered chloramphenicol was used for patients suffering from allergy, those having side effects (vomiting or profuse diarrhea), fever that persisted for > 5 days or relapse after therapy with macrolides. Antibiotic treatment prompted a clinical response in the shape of defervescence and an improvement in the clinical profile. Defervescence time was defined as the period from when antibiotic treatment began to the moment when defervescence first became constant (i.e. an axillary body temperature of ≤ 37°C for at least 3 consecutive days). Relapse was defined as the reappearance of fever within a week after the end of therapy.

### Statistical analysis

Analysis was carried out using the parametric ANOVA test for continuous factors; simultaneous comparisons of several proportions were performed using chi square test.

## Results

A total of 415 patients were diagnosed with *Rickettsia conorii *infection during the study period. The annual incidence of MSF infection ranged from 29 cases in 2004 to 98 in 1999. Most of the cases (*n *= 334) occurred between July and September, one case occurred in December. The median age was 6 years (range: 1 month-15 years). Two-hundred and forty-five (59%) of the children were male. A history of a tick-bite was given in 142 (34.2%) cases. Direct contact with dogs was reported in 86 (20.7%) cases. Patients were seen to in the emergency department after a median of 3 days (range 1–9) of fever. Table 1 shows the main clinical characteristics of the 415 cases.

**Table 1:** Clinical findings in 415 children with Mediterranean spotted fever.

Clinical manifestations	No. (%) of cases
Fever	386 (93)
Rash	392 (94.5)
Tache noire	263 (63.4)
Local lymphoadenopathy	214 (51.6)
Headache	122 (29.4)
Arthralgia and myalgia	158 (38.1)
Arthritis	3 (0.7)
Gastrointestinal symptoms	116 (27.9)
Hepatomegaly and/or splenomegaly	273 (65.8)
Orchitis	1 (0.2)
Conjunctival hyperaemia	53 (12.8)
Meningism	3 (0.7)
Meningitis	1 (0.2)

With regard to exanthema it was observed in 392 (94.5%) patients and was typical (maculopapular spreading to the palm and soles) in 374 (95.4%) of the cases and atypical in 18 cases (4.6%). Among atypical exanthema, petechial or purpuric rash was observed in nine cases (2.3%), papulovesicular in eight (2%), both purpuric and vesicular in one case (0.2%). Two hundred and thirty six patients presented exanthema, fever and tache noire together. Among 23 (5.5%) children without exanthema, 14 had fever, tache noire and lymphadenopathy, 2 fever and tache noire, 2 tache noire and lymphadenopathy, 2 only tache noire, 1 fever and lymphadenopathy, 1 fever and at last 1 headache and vomiting. In all tache noire was observed in 63.4% patients; it was a solitary lesion in 257 patients, while six patients presented two or three lesions.

Arthralgia and/or mialgia generally affected the joints and the muscles of the lower limbs and only in three (0.7%) cases restricted children's mobility. One girl aged 14 years developed meningo-encephalitis.

As regards haematological parameters, leucopenia (≤ 5,000/mm^3^) was found in 114 (27.5%) patients, leucocytosis (≥ 10,000/mm^3^) in 46 (11.1%), thrombocytopenia (< 150,000/mm^3^) in 54 (13%) cases, thrombocytosis (> 400,000/mm^3^) in nine (2.2%) cases. AST and/or ALT level were abnormal (>50 IU/ml) in 87 (21%) cases. In one patient ALT was 489 IU/l and no other infection was documented. Urine analysis, performed in 212 patients, revealed erythrocyturia in 145 (68.4%) and proteinuria in 105 (49.5%) cases.

Indirect immunofluorescence to *Rickettsia conorii *was performed in 365 (87.9%) cases. Serological confirmation of infection was obtained for 232 (63.6%) patients; 154 patients had diagnostic antibody titre on admission, while 78 showed a 4-fold titre elevation after two weeks. Among 133 cases not serologically confirmed, 124 patients had negative serology on admission and did not came back after two weeks; in nine cases with negative serology both on admission and in convalescence the diagnosis has been made on the grounds of clinical and epidemiologic criteria.

With regard to therapy, chloramphenicol was used from the beginning in 107 children (87 orally and 20 intravenously); 230 children were given clarithromycin, 78 azithromycin. All drugs were well tolerated, without evidence of toxicity or major side-effects. Twenty-eight patients initially treated with macrolides had to change therapy to i.v. chloramphenicol because of occurrence of vomiting and/or diarrhoea (15 patients treated with clarithromycin and one treated with azithromycin), itching rash (one patient treated with clarithromycin), fever that persisted for > 5 days (two patients treated with clarithromycin and nine treated with azithromycin). A further 19 patients treated with clarithromycin developed irrelevant side effects which did not require discontinuation of therapy.

Defervescence time and the mean duration of hospitalization in the three different groups of treatment are shown in table 2. There was no difference in the defervescence time (p = 0.093); in contrast, hospitalization was significantly longer in the chloramphenicol group compared with clarithromycin group (p = 0.018). The course of the disease was favourable in all cases.

**Table 2:** Efficacy and tolerability of the different drugs used in 415 patients with Mediterranean spotted fever.

	Chloramphenicol n = 107	Clarithromycin n = 230	Azithromycin n = 78	p
Mean time to defervescence (h)	40.62	41.94	47.13	0.093
Hospitalization mean (days)	5.4	3.4	4.1	0.018
Prolonged fever / relapse (%)	0	2 (0.9)	9 (11.5)	< 0.01
Side effects requiring drug discontinuation (%)	0	16 (7)	1 (1.3)	< 0.01

## Discussion

MSF is an acute disease caused by *Rickettsia conorii*, which is transmitted to humans by the brown dog-tick *Rhipicephalus sanguineus*. Ticks are not only vectors, but also reservoirs of *Rickettsia *so that physical contact with dogs is not necessary. In fact in our case series only a few patients (20.7%) presented this risk factor.

In our area the disease affects individuals of both sexes and all ages, and, in contrast to what was reported by Anton et al. [15], no peak is found in the pediatric population [1].

Comparing the results of our study with previous ones, we found clinical features similar to those reported by other authors except for a lower incidence of headache, arthralgia and myalgia and a higher frequency of hepato-splenomegaly (table 1) [10,15-17]. Headache is one of the typical and more discomforting symptoms of the disease in adults [15] and its lower frequency in children certainly contributes to the milder clinical presentation in this age group.

In the last decade newly recognized tick-borne rickettsioses have been shown to be prevalent in Europe. In 1997 *Rickettsia slovaca*, transmitted by the *Dermacentor marginatus *tick was linked to the illness occurring especially in children < 10 years, during the autumn and winter and characterized by scalp lesions due to tick-bites and enlarged regional lymph nodes. This clinical syndrome was denominated TIBOLA (tick-borne lymphadenopathy) [18]. In our case series, eight patients with tache noire in the scalp and lymphadenopathy, six of them also showing fever, could fit the diagnosis of TIBOLA. Because of the scarce specificity of standard serological methods [18] we are not be able to make a definitive diagnosis that could be made using isolation or molecular biology to establish and clearly identify agents.

Severe involvement of the central nervous system has been reported in adults with *Rickettsia conorii *infection [19-22], but is extremely rare in children [23-25]. We report a case of meningoencephalitis in a 14-yearold girl. The patient came to our notice after nine days of high fever, headache, arthralgia with no history of tick-bite. On clinical observation there was no evidence of tache noire and only a few maculopapular elements, involving neither the palms nor the soles, were present. The patient had a severely-affected central nervous system with obtunded consciousness, facial nerve deficit and meningeal signs. Examination of cerebrospinal fluid did not reveal any significant findings. Magnetic resonance imaging of the brain showed contrast enhancement in the meninges. Indirect immunofluorescence to *Rickettsia conorii *was positive on admission and showed a 4-fold titre elevation after two weeks. Intravenous administration of chloramphenicol for 15 days led to the disappearance of symptoms.

Standard treatment for MSF has been tetracyclines or chloramphenicol [26]. According to the American Academy of Pediatrics tetracyclines are not indicated for the treatment of common infections in children younger than eight years of age. However doxycycline is recommended for treatment of Rocky Mountain spotted fever in children of any age [27]. The milder clinical presentation and the benign course of MSF allow us to utilize macrolides, which are often used to treat infections in children, that lack adverse effects and are effective *in vitro *against *Rickettsia conorii *[28-30].

Several randomized clinical trials have been performed to compare macrolides in the treatment of MSF [31-33]. More recently good *in vivo *results were obtained for the new macrolides, identifying clarithromycin as a valid alternative therapy to tetracyclines or chloramphenicol for children aged < eight years [13,14].

In our case series there were no differences in the three groups of treatment with regard to the defervescence time (p = 0.093); the incidence of relapses and of side effects requiring a change of therapy was quite low, even if significantly different for the three drugs (p < 0.01). On the other hand, the shorter hospitalization stay in the clarithromycin group (p = 0.018), good compliance observed both in patients treated with clarithromycin and azithromycin, together with the withdrawal of oral formulation of chloramphenicol, confirm the leading role of clarithromycin in the treatment of pediatric MSF.

## Conclusion

As already known, MSF confirms to be an acute disease with a benign course in pediatric age. Nevertheless sporadic severe clinical manifestation can be observed. Complete evaluation of epidemiological, clinical and serological criteria is necessary to establish a prompt diagnosis and safe therapy. Clarithromycin and azithromycin, because of the lack of adverse effects and a good compliance, can be considered a valid alternative therapy to tetracyclines or chloramphenicol in the treatment of pediatric MSF especially for children aged < eight years.

## Competing interests

The author(s) declare that they have no competing interests.

## Authors' contributions

All authors contributed equally to this work.

## Pre-publication history

The pre-publication history for this paper can be accessed here:



## References

[B1] Ministero della Salute. Bollettino Epidemiologico Nazionale. http://www.ministerosalute.it/promozione/malattie/bollettino.jsp.

[B2] Jensenius M, Fournier PE, Raoult D (2004). Rickettsioses and the international traveler. Clin Infect Dis.

[B3] Raoult D, Brouqui P, Roux V (1996). A new spotted fever group rickettsiosis. Lancet.

[B4] Raoult D, Berbis P, Roux V, Xu W, Maurin M (1997). A new tick-transmitted disease due to Rickettsia slovaca. Lancet.

[B5] Nilsson K, Lindquist O, Pahlson C (1999). Associaton of Rickettsia elvetica with chronic perimyocarditis in sudden cardiac death. Lancet.

[B6] Fournier PE, Grunenberg F, Jaulhac B, Gastinger G, Raoult D (2000). Evidence of Rickettsia Helvetica infection in humans, eastern France. Emerg Infect Dis.

[B7] Giammanco G, Mansueto S, Ammatuna P, Vitale G (2003). Israeli spotted fever Rickettsia in Sicilian Rhipicaphalus sanguineus tick. Emerg Infect Dis.

[B8] Bacellar F, Beati L, Franca A, Pocas J, Regnery R, Filipe A (1999). Israeli spotted fever rickettsia (Rickettsia conorii complex) associated with human disease in Portugal. Emerg Infect Dis.

[B9] Zhu Y, Fournier PE, Eremeeva M, Raoult D (2005). Proposal to create subspecies of Rickettsia conorii based on multi-locus sequence typing and emended description of Rickettsia conorii. BMC microbiology.

[B10] Cascio A, Dones P, Romano A, Titone L (1998). Clinical and laboratory findings of boutonneuse fever in Sicilian children. Eur J Pediatr.

[B11] De Sousa R, Nobrega SD, Bacellar F, Torgal J (2003). Mediterranean spotted fever in Portugal: risk factors for fatal outcome in 105 hospitalized patients. Ann N Y Acad Sci.

[B12] Raoult D, Tissot-Dupont H, Caraco P, Brouqui P, Drancourt M, Charrel C (1992). Mediterranean spotted fever in Marseille: descriptive epidemiology and the influence of climatic factors. Eur J Epidemiol.

[B13] Cascio A, Colomba C, Antinori S, Paterson DL, Titone L (2002). Clarithromycin versus Azithromycin in the treatment of Mediterranean spotted fever in children: a randomized controlled trial. Clin Infect Dis.

[B14] Cascio A, Colomba C, Di Rosa D, Salsa L, Di Martino L, Titone L (2001). Efficacy and safety of clarithromycin as treatment for Mediterranean spotted fever in children: a randomized controlled trial. Clin Infect Dis.

[B15] Antón E, Font B, Muñoz T, Sanfeliu I, Segura F (2003). Clinical and laboratory characteristics of 144 patients with Mediterranean spotted fever. Eur J Clin Microbiol Infect Dis.

[B16] Font B, Bella F, Espejo E, Vidal R, Muñoz T, Nolla M, Casagran A, Mercade J, Segura F (1985). Mediterranean spotted fever: a cooperative study of 227 cases. Rev Infect Dis.

[B17] Font B, Espejo E, Muñoz T, Uriz S, Bella F, Segura F (1991). Fiebre botonosa mediterránea. Estudio de 246 casos. Med Clin (Barc).

[B18] Raoult D, Lakos A, Fenollar F, Beytout J, Brouqui P, Fournier PE (2002). Spotless rickettsiosis caused by *Rickettsia slovaca *and associated with *Dermacentor *ticks. Clin Infect Dis.

[B19] Charra B, Berrada J, Hachimi A, Judate I, Nejmi H, Motaouakkil S A fatal case of Mediterranean spotted fever. Med Mal Infect.

[B20] Alioua Z, Bourazza A, Lamsyah H, Erragragui Y, Boudi O, Karouach K, Ghfir M, Mossedaq R, Sedrati O (2003). Neurological feature of Mediterranean spotted fever: a study of four cases. Rev Med Interne.

[B21] Parra-Martinez J, Sancho-Rieger J, Ortiz-Sanchez P, Peset V, Brocalero A, Castillo A, Lopez-Trigo J (2002). Encephalitis caused by *Rickettsia conorii *without exanthema. Rev Neurol.

[B22] Marcos Dolado A, Sanchez Portocarrero J, Jimenez Madridejo R, Pontes Navarro JC, Garcia Urra D (1994). Meningoencephalitis due to *Rickettsia conorii*. Etiopathological, clinical and diagnostic aspects. Neurologia.

[B23] Thijssen HS, Leroy PL, van't Hek LG, Hurkx GA (2004). An unsuspected imported disease: meningo-encephalitis contracted in Spain. Ned Tijdschr Geneeskd.

[B24] Gear JH, Wagner JM, Dyssell JC, Hulton SA, Wehde SD (1990). Severe tick-bite fever in young children. A report of 3 cases. S Afr Med J.

[B25] Texier P, Rousselot JM, Quillerou D, Aufrant C, Robain D, Foucaud P (1984). Mediterranean boutonneuse fever. Apropos of a fatal case in a newborn infant. Arch Fr Pediatr.

[B26] Feigin RD, Snider RL, Edwards LS, Feigin RD, Cherry JD (1992). Rickettsioses. Textbook of pediatric infectious diseases.

[B27] Peter G, American Academy of Pediatrics (2003). Rocky Mountain spotted fever. Report of the committee on infectious diseases.

[B28] Ives TJ, Marston EL, Regnery RL, Butts JD, Majerus TC (2000). In vitro susceptibilities of Rickettsia and Bartonella spp. to 14-hydroxy-clarithromycin as determined by immunofluorescent antibody analysis of infected VERO cell monolayers. J Antimicrob Chemother.

[B29] Ives TJ, Manzewitsch P, Regnery RL, Butts JD, Kebede M (1997). In vitro susceptibilities of *Bartonella henselae*, *B. quintana*, *B. elizabethae*, *Rickettsia rickettsii*, *R. conorii*, *R. akari*, and *R. prowazekii *to macrolide antibiotics as determined by immunofluorescent-antibody analysis of infected Vero cell monolayers. Antimicrob Agents Chemother.

[B30] Rolain JM, Maurin M, Vestris G, Raoult D (1998). In vitro susceptibilities of 27 rickettsiae to 13 antimicrobials. Antimicrob Agents Chemother.

[B31] Munoz-Espin T, Lopez-Pares P, Espejo-Arenas E, Font-Creus B, Martinez-Vila I, Traveria-Casanova J, Segura-Porta F, Bella-Cueto F (1986). Erythromycin versus tetracycline for treatment of Mediterranean spotted fever. Arch Dis Child.

[B32] Meloni G, Meloni T (1996). Azithromycin vs. doxycycline for Mediterranean spotted fever. Pediatr Infect Dis J.

[B33] Bella F, Font B, Uriz S, Munoz T, Espejo E, Traveria J, Serrano JA, Segura F (1990). Randomized trial of doxycycline versus josamycin for Mediterranean spotted fever. Antimicrob Agents Chemother.

